# Primary Prevention of Stroke in Children With Sickle Cell Anemia in Nigeria: Protocol for a Mixed Methods Implementation Study in a Community Hospital

**DOI:** 10.2196/37927

**Published:** 2022-06-13

**Authors:** Halima Bello-Manga, Lawal Haliru, Kudrat Abdulkareem Ahmed, Abdulkadir Musa Tabari, Bilkisu Usman Farouk, Gloria Yimi Bahago, Aisha Shuaibu Kazaure, Abdulrasheed Sani Muhammad, Samira Abubakar Gwarzo, Ana A Baumann, Michael R DeBaun, Allison A King

**Affiliations:** 1 Department of Hematology and Blood Transfusion Barau Dikko Teaching Hospital/Kaduna State University Kaduna Nigeria; 2 Department of Pediatrics Barau Dikko Teaching Hospital/Kaduna State University Kaduna Nigeria; 3 Department of Radiology Barau Dikko Teaching Hospital/Kaduna State University Kaduna Nigeria; 4 Department of Nursing Services Haematology Unit Barau Dikko Teaching Hospital Kaduna Nigeria; 5 Department of Pharmaceutical Services Barau Dikko Teaching Hopsital Kaduna Nigeria; 6 Research Office Barau Dikko Teaching Hospital Kaduna Nigeria; 7 Department of Surgery Washington University in St. Louis St. Louis, MO United States; 8 Department of Pediatrics Division of Pediatric Neurology Vanderbilt University School of Medicine Nashville, TN United States; 9 Program in Occupational Therapy Department of Medicine, Pediatrics, Surgery, and Education Washington University School of Medicine St. Louis, MO United States

**Keywords:** sickle cell anemia, stroke prevention, transcranial Doppler ultrasonography

## Abstract

**Background:**

In Nigeria, approximately 150,000 children with sickle cell anemia (SCA) are born annually, accounting for more than half of all SCA births worldwide. Without intervention, about 11% of children with SCA will develop a stroke before their 20th birthday. Evidence-based practices for primary stroke prevention include screening for abnormal transcranial Doppler (TCD) measurements coupled with regular blood transfusion therapy for at least one year, followed by hydroxyurea (HU) therapy indefinitely. In high-resource countries, this strategy contributes to a 92% decrease in stroke incidence rates. In 2016, as part of a capacity building objective of the Stroke Prevention Trial in Nigeria (1R01NS094041: SPRING), TCD screening was adopted as standard care at Barau Dikko Teaching Hospital in Kaduna. However, with just 70 radiologists and only 3 certified in TCD screening in the state, just 5.49% (1101/20,040) of eligible children with SCA were screened. Thus, there is a need to explore alternate implementation strategies to ensure children with SCA receive standard care TCD screening to decrease stroke incidence.

**Objective:**

This protocol describes a study to create a stroke prevention program in a community hospital in Kaduna through task shifting TCD screening to nurses and training medical officers to initiate and monitor HU utilization for stroke prevention.

**Methods:**

This study will be conducted at 2 sites (teaching hospital and community hospital) over a period of 3 years (November 2020 to November 2023), in 3 phases using both quasi-experimental and effectiveness-implementation study designs. In the needs assessment phase, focus groups and structured interviews will be conducted with health care providers and hospital administrators to identify barriers and facilitators to evidence-based stroke prevention practices. Results from the needs assessment will inform intervention strategies and a process plan to fit the needs of the community hospital. In the capacity building phase, nurses and medical officers at the community hospital will be trained on TCD screening and HU initiation and monitoring. In the implementation phase, children with SCA aged 2-16 years will be recruited into a nonrandomized single-arm prospective trial to determine the feasibility of initiating a task-shifted stroke prevention program by recording recruitment, retention, and adherence rates. The Reach and Effectiveness components of the RE-AIM (Reach, Effectiveness, Adoption, Implementation and Maintenance) framework will be used to evaluate implementation outcomes between the community and teaching hospitals.

**Results:**

The needs assessment phase of the study was completed in February 2021. Manuscript on findings is currently in preparation. Capacity building is ongoing with TCD training and sickle cell disease and stroke education sessions for nurses and doctors in the community hospital. Recruitment for the implementation trial is expected to commence in July 2022.

**Conclusions:**

This study proposes a structured, theory-driven approach to create a stroke prevention program in a community hospital in Kaduna, Nigeria, to decrease stroke incidence among children with SCA. Results will provide preliminary data for a definitive randomized clinical trial in implementation science.

**International Registered Report Identifier (IRRID):**

PRR1-10.2196/37927

## Introduction

### Background

Sickle cell anemia (SCA) is the leading cause of stroke in children globally. Without any intervention for primary stroke prevention, children with SCA have a 10% annual risk of developing a stroke [1]. In sub-Saharan Africa, about 11% of these children will develop a stroke before their 20th birthday [2]. In Nigeria, the country with the highest birth rate of newborns with SCA, over 150,000 births per year, there is a minimum estimate of 10,000 stroke episodes in children with SCA for the birth year cohort (assuming minimum mortality) [3]. In high-income countries, evidence-based practices for primary stroke prevention in children with SCA involve screening for abnormal transcranial Doppler (TCD) ultrasound velocity coupled with regular blood transfusion therapy for at least one year followed by hydroxyurea (HU) therapy indefinitely [4,5]. This strategy has decreased the risk of stroke by 92%, leading to a 10-fold drop in stroke incidence from 0.67 to 0.06 strokes per 100 patient-years [6,7]. Unfortunately, well-established evidence-based practices for SCA have not yet been transferred to low-resource settings such as Nigeria. There exists a gross paucity of TCD services in Nigeria, largely due to a lack of trained personnel certified in performing TCD and a shortage of TCD machines dedicated to primary stroke prevention [8,9]. In Nigeria, TCD ultrasonography training is primarily restricted to radiologists.

In 2012, a feasibility trial to determine the acceptability of HU therapy for primary stroke prevention in children with abnormal TCD measurements was conducted in Kano, Nigeria (SPIN [Stroke Prevention in Nigeria]: NCT01801423). The team demonstrated high participant recruitment, retention, and adherence rates for HU as primary stroke prevention in children with SCA living in northern Nigeria and established standard care for stroke prevention using HU at 20 mg/kg/day [9]. Further, a multicenter trial (SPRING [Primary Stroke Prevention in Nigeria]: NCT02560935) was completed in 3 centers in 2 states, Kano and Kaduna in northern Nigeria, to answer a critical question on the optimal dose of HU (20 mg/kg vs. 10 mg/kg) for primary stroke prevention. Results demonstrated that in low-income settings without access to indefinite regular blood transfusion therapy, fixed low-dose HU of at least 10 mg/kg/day is effective for primary stroke prevention [10]. More recently, the 2020 American Society of Hematology guidelines on central nervous system complications in sickle cell disease (SCD) recommend that children with SCD and abnormal TCD measurements living in low- and middle-income countries where regular blood transfusions are not readily available receive at least moderate dose (20 mg/kg/day) of HU [11].

The earlier trials (SPIN and SPRING) were all conducted in academic hospitals with the right combination of staff and facilities and, suffice to say, research and implementing findings into usual care may be more feasible in such resource-rich centers. To achieve equitable translation of “research to practice,” there is a need to include community hospitals in the conduct of research. For us to rapidly translate the findings from our Stroke Prevention Trial, we propose transporting evidence-based practices for stroke prevention from our trial site, Barau Dikko Teaching Hospital (BDTH), a 300-bed academic hospital located in Kaduna North Local Government, to Yusuf Dantsoho Memorial Hospital (YDMH), a 100-bed community hospital located in Kaduna South Local Government run by the State’s Ministry of Health. The premise of this feasibility trial is that improving access to TCD screening in a community hospital will dramatically increase the rate of TCD screening, thereby leading to a decrease in stroke occurrence among children with SCA.

Our prior findings indicate that physician-only TCD screening is insufficient to increase the reach of TCD screening for stroke prevention among children with SCA living in Kaduna or the rest of Nigeria. Of the approximately 20,040 children aged 0-14 years with SCD eligible for TCD screening in Kaduna metropolis, only 5.49% (n=1101) have ever had a TCD screening in their lives [12,13]. Prior to the Stroke Prevention Trial, no TCDs were performed in the state owing to a lack of trained personnel and absence of TCD machines. Currently, there are approximately 300 radiologists available in Nigeria serving a population of nearly 175.5 million, an estimate of 1 radiologist per 658,000 people [14,15]. Given the paucity of radiologists in the region, an alternate implementation strategy focused on training nurses in community hospitals to perform TCD screening will dramatically increase the number of eligible children who will have access to stroke prevention services. Similarly, training the medical officers already working in the community hospital on initiation and monitoring of HU administered to children with abnormal TCD measurements will be far more efficient and sustainable than full dependence on the insufficient numbers of specialists and subspecialists (pediatricians or hematologists). Additionally, significantly more people attend the community hospital (YDMH) because of its location in a more densely populated area of the metropolis and lower cost compared with the teaching hospital (9214 people/km^2^ vs. 6835 people/km^2^ and US $4.58/outpatient visit vs. US $6.77/outpatient visit, respectively) [16].

### Rationale for Task Shifting

The World Health Organization (WHO) defines task shifting as “the rational redistribution of tasks among health workforce teams. Specific tasks are moved, where appropriate, from highly qualified health workers to health workers with shorter training and fewer qualifications in order to make more efficient use of available human resources for health” [17]. In Nigeria, as with many other resource-constrained countries, there is a shortage of well-trained health workers [18]. Currently, WHO estimates 4 doctors per 10,000 population in Nigeria compared with 26 doctors per 10,000 population in the United States [19,20]. Additionally, the available health workers are unevenly distributed, with a higher concentration in urban areas and private sectors [18,21]. Even if Nigeria embarks on an emergency training of doctors equipped to offer stroke prevention to children with SCA, it will take 6 years to certify generalists and another 4 years to train a specialist (radiologist or hematologist). Clearly, other alternatives are needed to address this shortage of qualified health personnel. Therefore, task shifting “presents a viable solution for improving health care coverage by making more efficient use of human resources already available and by quickly increasing capacity while training and retention programs are expanded” [17]. Task shifting can produce equivalent or superior outcomes for many diseases and health interventions [22].

### Objectives

The overall goal of this protocol is to create a stroke prevention program in a community hospital (YDMH) through task shifting of TCD screening to nurses and initiation of HU to nonspecialist medical officers. This strategy will help in translating research to usual care by (1) reaching a larger number of children with SCA eligible for stroke screening and (2) identifying those with abnormal TCD values who will benefit from stroke prevention with HU, thereby decreasing stroke occurrence.

The specific objectives of this protocol are to

1. Identify barriers and facilitators that influence the adaptability of the transported evidence-based practice intervention, including the implementation process;
2. Build capacity for stroke detection and prevention in SCA at a community hospital; and
3. Conduct a feasibility trial comparing the effectiveness of a physician-based primary stroke prevention program at an academic site with a task-shifted primary stroke prevention program at a community site.

## Methods

### Study Design and Setting

This study will be conducted at 2 sites, BDTH academic hospital and YDMH community hospital, using both quasi-experimental (objectives 1 and 2) and effectiveness-implementation (objective 3) designs. Procedures will be conducted in 3 phases: (1) needs assessment, (2) capacity building, and (3) feasibility of the implementation trial.

### Phase 1: Needs Assessment

#### Objective

The goal of this phase is to better understand the current knowledge and perception of health care providers and hospital administrators on stroke in SCA to inform future interventions in the community hospital using a prospective, qualitative study design.

#### Recruitment, Eligibility, and Stratification

Nonspecialist physicians and nurses working in the pediatric unit at the YDMH community hospital will be recruited to participate in a series of focus groups, after an advocacy visit and permission is received from the hospital management. These providers run a weekly SCD clinic with an average attendance of 50 patients per week. We will use a purposeful recruitment approach to maximize input from participants on the needs assessment [23]. Per best practices, multiple focus groups will be planned from different perspectives (samples) to triangulate issues and concerns. We will stratify within each sample and conduct several focus groups per stratum [24,25]. We anticipate a total of 4 focus groups per sample (comprising 4-6 participants each) among nurses and doctors. We anticipate that 2 focus groups per stratum will be adequate for saturation. If needed, we will conduct a third group in each stratum.

We will also conduct informant interviews for key hospital administrators, notably the Medical Director, Director of Administrator, Chief Matron, and Chief Accountant of the Community Hospital.

#### Focus Group and Interview Procedures

Following individuals’ consent to participate and self-reported eligibility confirmation, the research coordinator will convene focus groups at times convenient for participants. Providers will receive a reminder phone call or SMS text message 48 hours before the scheduled focus group to maximize attendance. Focus groups will be led by a trained facilitator and will last about 45 minutes. During the focus group, the principal investigator (PI) will provide supplemental education on SCD, and participants will be provided with lunch. Focus groups will be audiotaped, transcribed, and deidentified. During the focus group, a research assistant will take field notes to supplement the transcript. Throughout the needs assessment phase, the PI and other senior key personnel will meet weekly to assess fidelity, focus group processes and progress, discuss emerging themes, and assess progress toward saturation. Interviews with hospital administrators will be scheduled at times convenient for the individual and last about 30 minutes.

**Figure 1:**
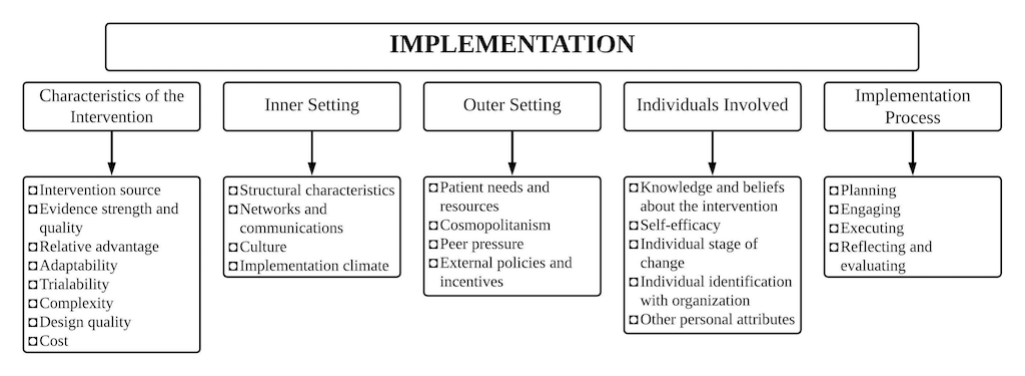
Consolidated framework for implementation research.

Facilitators of the focus groups and interviews will use a structured guide, including moderator instructions, key questions, and suggested probes, developed by the PI and mentors (see Multimedia Appendices 1 and 2). The focus group and interview guides, informed by the Consolidated Framework for Implementation Research (CFIR), will address 5 domains of implementation (Figure 1). Textbox 1 presents sample questions. Similar questions will be asked for each stratum with minor wording changes where appropriate. The semistructured interview conducted with hospital administrators will mainly address barriers and facilitators to the implementation process.

**Consolidated Framework for Implementation Research domains to be addressed by focus groups and interviews.**

**Intervention Characteristics (Adaptability)**

*Health Care Providers*

Do you think it is possible to task shift the conduct of transcranial Doppler (TCD) and stroke detection to nurses?

- What role do you think nonphysicians should play in stroke prevention in children with sickle cell disease (SCD)?
- Tell me how you feel about nonphysicians doing TCD screening and stroke detection
- Tell me how you feel about task shifting

*Administrators*

How do you feel about task shifting?

- How do you think task shifting should be done in your organization?
- Who do you think should be involved in task shifting?

**Characteristics of the Individual (Knowledge and Belief About the Intervention and Self-efficacy)**

*Health Care Providers*

What do you know about SCD and stroke?

- Tell me how you think a child with stroke will look like
- How do you think you can recognize a child with stroke?

Do you think you can help prevent stroke in children with SCD?

- In what ways do you think you can help?
- Tell me what will you do
- Tell me the skills you would like to have for you to help prevent children from having a stroke

*Administrators*

Do you know about SCD and stroke?

- How do you think stroke affects children?
- How do you think children can be prevented from having a stroke?
- Do you think children can have a stroke?
- Do you think children that had a stroke will recover completely?
- How do you think you can help them?

**Outer Setting (Patient Needs and Resources)**

*Health Care Providers*

Do you think you need more information on SCD and stroke?

- Tell me what you would like to know about SCD
- How would you like the information to be provided?

*Administrators*

Tell me the kind of information you would like to have on SCD

- How would you like the information to be provided?

**Inner Setting (Implementation Climate)**

*Health Care Providers*

How do you think task shifting will fit into your current schedule?

- How do you feel about task shifting?
- Tell me how you think you will adapt
- Tell me how you will help in making task shifting efficient

*Administrators*

How will you implement task shifting?

- Tell me how you will introduce task shifting
- Tell me how you will ensure that task shifting is sustained

**Implementation Process (Planning and Engaging)**

*Health Care Providers*

How do you intend to inform parents about the stroke prevention program?

- Tell me the ways that you will ensure parents bring their children for TCD and stroke detection
- How will you follow children to ensure they have their TCD at the scheduled times?

How will you get the buy-in of parents/caregivers for the program?

- Tell me how you will engage parents/caregivers to advocate for TCD screening in the community
- Tell me how you will communicate with them

*Administrators*

How do you intend to start the stroke prevention program?

- Tell me how you will ensure your staff supports the program
- Tell me how you will ensure the program is continued

How will you get the buy-in of staff within your organization?

- Tell me how you will identify champions within the organization

Tell me how you intend to engage them

We will also administer a brief (<10 minutes) prediscussion survey before each focus group and interview (Multimedia Appendices 1 and 2). For nurse and doctor participants, we will query age, gender, ethnicity, job description/title, patient population, percentage of patients with SCD, and years of experience caring for individuals with SCD. For hospital administrator participants, we will query age, gender, job description, average cost of hospital admission, approximate number of individuals with SCD attending the hospital annually, role in redistribution of staff within the hospital, and about 5 questions on SCD complications and the approximate cost of care.

#### Data Analysis

Qualitative data will be analyzed based on principles from grounded theory using both inductive and emergent coding strategies and methods used in previous studies [26-28]. Coding will begin with open coding of the transcript to identify topics and codes to develop a codebook. This will be followed by thematic coding to identify common categories and meanings to reach thematic saturation (ie, no new themes will be captured with further analysis). Primary and secondary coders will develop a codebook of themes to complete analysis. Removing identifiers from transcripts will preserve participants’ confidentiality. We will also remove any participants’ reports of unusual circumstances that could make them identifiable.

### Phase 2: Capacity Building

#### Objective

The goal of this phase is to address the barriers to stroke prevention identified in the needs assessment phase by providing education and training using a pre- and poststudy design. We will apply a previously established education program for stroke detection in children on nurses and medical officers at the community hospital [29]. We will also train nurses at the community hospital to conduct TCD screenings through a previously established TCD certification program. To guide the application of these already established educational and training materials [29], we will use our findings from the needs assessment phase, guided by the CFIR framework (Figure 1), and focus particularly on the “characteristics of the individuals” domain to address the “knowledge and beliefs about the intervention” construct.

#### Recruitment and Eligibility

We will use convenience sampling for the conduct of this phase, and participants will be selected based on availability and willingness to participate [23]. Four nurses working in the pediatric clinic at YDMH community hospital will be approached for training on stroke detection and TCD screening. Based on interest, 2 nurses will be selected for training. Reasons for interest and disinterest will be documented. All medical officers working in the pediatric clinic will be approached for participation. Two medical officers will be recruited based on interest. Children with SCD meeting the eligibility criteria for TCD screening will be recruited for training purposes via the weekly pediatric SCD clinic. The recruitment and training will be done over a period of 6 months.

#### Training Procedures and Outcome Measures

Following consent, nurses and medical officers will be enrolled in an educational course, which will emphasize detecting strokes and common clinical problems seen in children with SCA. A 20-minute video in Hausa (native language of most individuals that live in Kaduna, Nigeria), developed with members of our team, has already been created to reinforce the common causes of morbidity in children with SCD, including fever management, splenic sequestration, vaso-occlusive pain episodes, and stroke. This video will be used to support didactic lectures and will serve to augment the key learning objectives expected in the courses.

For stroke scale certification, nurses will be taught to perform the Pediatric National Institutes of Health Stroke Scale (PedNIHSS), a validated, standardized neurological examination used in previous trials [9,30]. Nurses will watch a set of 5 videos created by a certified neurologist, designed to instruct nonphysicians on how to conduct a pediatric neurological examination as per the PedNIHSS. Topics include (1) stroke in SCD, (2) pediatric neurology history and examination, (3) neurologic history and examination findings that suggest a child has had a stroke, (4) the PedNIHSS, and (5) supportive care immediately after a stroke has occurred. Similarly, educational materials describing SCD, mode of transmission, and some complications including symptoms of stroke, for example, FAST (Face drooping, Arm weakness, Speech difficulty, and Time to call for help), already being utilized at the teaching hospital (BDTH) will be shared with health providers at the YDMH community hospital for patient education during weekly health talks. A card describing the FAST acronym with pictograms and simple, concise language (both English and Hausa) will be provided and shared with patients to take home with them. We will administer a pre- and posttest after each health education session to assess for changes in knowledge among participating nurses.

The Stroke Prevention Trial protocol will be used to train nurses on TCD screening [7]. One of the 2 original Nigerian TCD-certified radiologists will implement the preexisting TCD screening training program, which covers didactic theory lectures and practical aspects of TCD measurements for a minimum of 60-90 hours for 2 weeks. All TCD training and examinations will be done on the same nonimaging TCD machine (Lucid M1 Systems; Neural Analytics Inc.). The research team will interview participating nurses during and at the end of the training to capture challenges, feasibility, and acceptability of the training program. Feedback from nurses will be used to adapt the training for future trials.

#### Data Analysis

To pass the stroke scale certification training, nurses will be required to pass the online NIH (National Institutes of Health) Stroke Scale Certification, which requires a total of 84 correct answers out of 90 in each of the 6 sections of training [31].

Nurses will obtain TCD certification based on Spearman rank correlation (r) between the trainer and nurse trainees. As per the protocol in our Fogarty-NINDS–funded R21 and R01 randomized controlled primary stroke prevention trials, each trainee and trainer will have at least 40 paired TCD measurements as part of their TCD certification examination, with at least four children that have prior documented abnormal TCD measurements. Passing the TCD certification will require a correlation between the trainee and trainer exceeding the lower bound of 0.76 (ie, 85% of the documented trainer’s correlation as the minimum acceptable correlation threshold) [32]. The data will be analyzed using R Core (2016) [33].

### Phase 3: Feasibility of the Implementation Trial

#### Objective

The goals of this phase are to (1) conduct a nonrandomized single-arm prospective trial to determine the feasibility of initiating a stroke prevention program for children with SCA in a community hospital (YDMH) through “task shifting” by recording recruitment, retention, and adherence rates; and (2) conduct an effectiveness-implementation study to test the hypothesis that a “task-shifted” approach for primary stroke prevention in a community hospital demonstrates noninferior effectiveness in identifying children with abnormal TCD and stroke compared with a physician-led primary stroke prevention at a teaching hospital.

#### Recruitment and Eligibility

We will use convenience sampling, and participants will be selected based on availability and willingness to participate [23]. Children meeting the following eligibility criteria will be recruited via pediatric SCD clinics at both BDTH (teaching hospital) and YDMH (community hospital): (1) diagnosis of SCA (hemoglobin [Hb] SS or Sβthal^0^), and (2) between 2 and 16 years of age. Children will be excluded from participation if they meet any of the following criteria: (1) prior overt stroke (a focal neurological deficit of acute onset) by history, focal neurological deficit on standardized neurological examination, or concern for moderate or severe neurological deficit (which could be due to stroke); (2) already on HU therapy either for stroke prevention or for other indication; (3) prior participation on the therapy arm of the SPRING trial [34]; (4) have comorbid conditions such as asthma or epilepsy; and (5) have other exclusions for HU including significant cytopenia (Hb <6 g/dL, absolute neutrophil count <1.5 × 10^9^/L, platelets <150,000/µL, reticulocytes <80,000/µL [unless Hb is >9 g/dL], renal insufficiency [creatinine >0.8 mg/dL]). The study will be introduced to patients during the routine “health talk” given in the morning of every clinic, and informational leaflets and flyers will be provided. Informed consent will be obtained from a legal guardian for all participants. Assent will be obtained from participants aged 5-16 years. Enrolled participants will be followed for 12 months.

#### Feasibility Trial Procedures and Outcome Measures

Consenting participants will undergo TCD screening and neurological examination for stroke detection. Participants’ medical history will be taken and physical examination conducted. Blood and other biological samples will be drawn for laboratory investigations.

If the participant has elevated TCD levels (≥200 cm/second on 2 consecutive measurements or a single measurement ≥220 cm/second), they will be offered blood transfusion first as the default standard care in primary stroke prevention in SCA. If the participant and family elect not to receive blood transfusions, they will be invited to participate in the study. The participant will complete the consent and assent process for study screening and evaluations as outlined in Table 1. If the participant and family elect to participate, the participant must successfully swallow a pill (vitamin C tablets) in the presence of study personnel prior to being allocated to receive study therapy. If successful, study personnel will review the schedule of study procedures with the participant and family as shown in Table 2. For children with conditional TCD measurements (>180 and <200 cm/seconds), we recommend that follow-up TCD measurements be done as per routine clinical practice, but typically a child with a conditional TCD velocity would have a repeat TCD measurement within 3 months, and often sooner.

Both study sites will complete laboratory monitoring to identify potential adverse events or complications associated with SCD and being on HU therapy. A local monitor based at the site will review all laboratory values weekly. Based on the laboratory values of the SPIN Trial (NCT01801423), we have been able to select the 10th and 90th percentile for each laboratory value that will be assessed. We have also established decision rules for each laboratory value to guide health care providers on how to monitor for potential toxicities (Table 3). In circumstances where laboratory monitoring cannot be completed at the study site due to institution or local strike, it will be conducted at a back-up laboratory.

**Table 1:** Screening and initial evaluation.

Procedure		Visit 1
**Initial review for study screening**		
	Informed consent and assent for study screening	✓
	History and physical examination	✓
	CBC/diff^a^, reticulocytes	✓
	Hb^b^ F and S quantification	✓
	Urinalysis	✓
	Urea/electrolyte/creatinine	✓
	Liver function test	✓
	Confirm eligibility for study screening based on laboratory values	✓
**Continued initial review for study screening for participants with Hb greater than 6 g/dL**		
	Baseline Stroke-Free Questionnaire	✓
	Baseline PedNIHSS^c^ neurological evaluation	✓
	TCD^d^	✓
	Capsule swallowing confirmation by research personnel	✓
	Pregnancy test (same time as urinalysis when menses is reported)	✓
	Confirm eligibility for study therapy	✓
	Obtain informed consent and assent for study therapy	✓

^a^CBC/diff: complete blood count with differential.

^b^Hb: hemoglobin.

^c^PedNIHSS: Pediatric National Institutes of Health Stroke Scale.

^d^TCD: transcranial Doppler.

**Table 2:** Schedule of study procedures.

Procedure	Interim visits for HU^a^ therapy (months 1-12)	Month 12
Informed consent and assent for study therapy	Reconsent as needed	Reconsent as needed
History and physical examination	✓	✓
TCD^b^	Every 3 months	✓
Follow-up Stroke-Free Questionnaire	✓	✓
Study medication refill/pill counting	✓	✓
Adherence intervention	✓	✓
CBC/diff^c^, reticulocytes	✓	✓
Urinalysis	✓	✓
Urea/electrolyte/creatinine	✓	✓
Liver function test	✓	✓
10 Questions neurological screening	✓	✓
Annual PedNIHSS^d^ neurological evaluation	✓	✓

^a^HU: hydroxyurea.

^b^TCD: transcranial Doppler.

^c^CBC/diff: complete blood count with differential.

^d^PedNIHSS: Pediatric National Institutes of Health Stroke Scale.

**Table 3:** Laboratory monitoring of HU.

Laboratory parameters and when to stop therapy		Remarks
**Hb^a^**		
	Hb <6 g/dL Give blood transfusion if Hb <5 g/dL Resume HU^b^ if Hb >6g/dL	Evaluate for nutritional deficiency (Fe, B12, folate). If iron deficiency is suspected, give FeSO4 at 3 mg/kg Investigate for other causes (bleeding, helminthiasis, malaria, renal diseases)
**Platelet count**		
	Platelet <80 × 109/L Resume HU if platelets >80 × 109/L	Repeat CBC^c^ on the same day or within a week. Investigate for viral infections.
**ANC^d^**		
	ANC <1000 × 109/L Resume HU if ANC >1000 × 109/L	Stop HU and repeat CBC weekly until ANC >1000 × 109/L. If ANC <1000 × 109/L after 2 weeks, refer to the consultant pediatrician. Once ANC is >1000 × 109/L, restart HU at a dose decreased by 20%. Repeat CBC after 2 weeks of change in dose.
**Reticulocyte count (not available routinely)**		
	Reticulocyte count <1% and Hb <6 g/dL	Repeat reticulocyte and Hb measurements in 1 week. Evaluate for other causes (bone marrow disorder, nutritional deficiencies [Fe, B12, folate]). Refer to a consultant to treat underlying cause.

^a^Hb: hemoglobin.

^b^HU: hydroxyurea.

^c^CBC: complete blood count.

^d^ANC: absolute neutrophil count.

In an effort to maximize adherence to HU therapy, we have developed a *Parent Handbook* that is part of standard care for children on HU therapy and has been translated into Hausa. The Handbook emphasizes the following:

- The key to the success of retention and follow-up will be attention to detail; consistency of the research team; and trust among the parents, patients, and staff. The staff will contact parents immediately and reschedule patients’ appointments when missed.
- Close monitoring of participants’ visits and need for return appointments with follow-up telephone calls are key elements for monitoring patient attendance and prevention of study participants’ being lost to follow-up. Every attempt will be made by the study team to contact patients prior to their scheduled visit based on the allocated schedule for participants on HU therapy.
- Identification of a stable contact person (such as a grandparent, neighbor, teacher, friend) who does not live with the participant’s family, but always knows the family’s whereabouts will assist in tracking patients and decrease the number lost to follow-up.
- Additionally, the site will log patients who are approached to participate in the study and document whether the patient and family elects or declines involvement with the study. The rationale for not participating in the trial will be inclusive of but not limited to time commitment for the study, therapy regimen, adherence to HU therapy, etc.

All participants will be followed for up to 12 months to assess rate of recruitment, retention, and adherence to the treatment protocol for primary stroke prevention for those with abnormal TCD measurements. *Recruitment rate* is defined as the proportion of participants who agreed to participate in the study out of the total number of persons approached. For this study, we anticipate a minimum recruitment rate of 80% based on our team’s experience with the SPIN trial in Kano, Nigeria [9]. *Retention rate* is defined as the percentage of study participants who completed the study protocol at the end of 12 months. We expect a retention rate of greater than 80%. *Adherence rate* is defined as the proportion of children with laboratory evidence of sustained HU therapy (primary measure), measured by an increase in mean cell volume of at least 10 fL from baseline, and by the percentage of pills returned from the total amount administered to the pharmacy every 3 months (secondary measure). We expect a therapy adherence rate for both primary and secondary measures of at least 80% (eg, 80% of patients will have an increase of at least 10 fL in mean corpuscular volume from baseline [9]).

We will use the “Reach and Effectiveness” components of the RE-AIM (Reach, Effectiveness, Adoption, Implementation and Maintenance) framework to evaluate implementation outcomes between the community and teaching hospitals [35,36]. The number of eligible children with SCA that have TCD screening in both the teaching and community hospitals will measure the “Reach”; while “Effectiveness” will be measured by the proportion of children with abnormal TCD measurement started on HU (averting stroke) between the academic and community site (primary outcome). Secondary outcomes will include (1) incidence of other clinical events including pain crises, fever, acute chest syndrome, or severe anemia in children with abnormal TCD measurements receiving HU in both the academic and community sites, (2) incidence of clinical events among children with SCA not receiving HU, and (3) death.

#### Sample Size Determination

For the feasibility trial, our focus is whether or not this trial is feasible based on rates of recruitment, retention, and adherence to trial therapy. We will use a CI approach to ensure that we can achieve a minimal level of recruitment, retention, and adherence rates to trial medication (HU) that would be acceptable. If we do not achieve this minimum rate, we will examine other alternative approaches and barriers needing to be resolved before proceeding with a full-scale trial. In this case, we consider compliance is dichotomous (yes or no). Thus, we can assume a binomial distribution, and calculate corresponding 95% CIs. Because we are only focused on ensuring a floor level of compliance, where we define compliance as an increase in mean corpuscular volume ≥10 fL, we will use 1-sided CIs (ie, ensure lower bound is above our lowest acceptable rate of 75%). With a sample size of 100, and observed compliance rates of 83% and higher, using the Clopper-Pearson formula, we will obtain a lower bound of 75.6% or greater.

### Data Analysis

We will use proportions and CIs to calculate the percentages of participants who have agreed to be part of the trial, adhere to the trial drug (HU), and complete the 1-year follow-up. Person times will be calculated to determine incidence rate ratio of stroke. We will use Cox regression and the Kaplan-Meier plot to calculate the hazard rate for stroke recurrence and probability of recurrence.

Quantitative data will be analyzed using descriptive, comparative, and correlational statistics to measure the implementation outcomes of Reach and Effectiveness before and 12 months after intervention.

### Ethics Approval

The ethics committee of the Kaduna State Ministry of Health and BDTH/Kaduna State University approved this protocol on June 9, 2020, and June 22, 2020, respectively.

## Results

Funding for the study is from the Fogarty International Center and National Institute of Neurological Disorders and Stroke (K43TW011583). The Institutional Review Board of BDTH and Kaduna State Ministry of Health approved the study. The needs assessment phase of the study was completed in February 2021. Manuscript on findings is currently in preparation. Capacity building is ongoing with TCD training and SCD and stroke education sessions for nurses and doctors in the community hospital. Recruitment for the implementation trial is expected to commence in July 2022.

## Discussion

### Principal Findings

This study proposes a structured, theory-driven approach to create a stroke prevention program in a community hospital in Kaduna, Nigeria, to decrease stroke incidence among children with SCA. Results will provide preliminary data for a definitive randomized clinical trial in implementation science.

We hypothesize that establishing a stroke prevention program in a community hospital by task shifting of TCD screening to nurses and administration of HU to children with abnormal TCD values to medical officers will increase the number of children with SCA identified with the risk of stroke, leading to a decrease in stroke incidence. Because of the lack of skilled personnel, stroke prevention programs are only available, prior to this study, at academic hospitals where there are many specialists that offer these services. Our task-shifting strategy will address the gap in the available human resources at the community level by building the capacity of nurses and medical officers.

### Comparisons With Prior Work

We have previously established stroke prevention programs in academic hospitals following the successful completion of the SPIN (NCT01801423) and SPRING (NCT02560935) trials in Nigeria. Given the paucity of radiologists, nonspecialist medical officers were trained to conduct TCD screening in our previous trials. However, we figured this was not a very sustainable strategy because after sometime, these medical officers go on to start their specialist training. Therefore, to ensure sustainability, we chose to train nurses in the community hospital on the conduct of TCD. This way, children will be seen and have their TCD done on the same day and those that have abnormal TCD values can be started on HU.

### Strengths and Limitations

The strength of our study lies in the mixed method design we are using. The qualitative aspect gives us an insight into the context of the community in which the intervention is being introduced. We will be able to identify the barriers and facilitators to the intervention and tailor them toward implementation and sustainability. Additionally, using quantitative methods will allow us to measure the actual impact of the stroke prevention program. A weakness we have identified is the cost associated with the multiple trainings that nurses and medical officers have to undergo and our inability to address some of the identified barriers, which may be out of our control.

### Future Directions

This study will set the stage for further scaling-up stroke prevention services that will be accessible to eligible children with SCA living in low-middle income countries like Nigeria. Further, the approach of conducting TCD screening and initiation of HU on the same day can serve as a basis for “reverse innovation” in other resource-rich countries where the uptake of TCD screening is suboptimal.

### Conclusion

This study proposes a structured, theory-driven approach to create a stroke prevention program in a community hospital in Kaduna, Nigeria, to decrease stroke incidence among children with SCA. Results will provide preliminary data for a definitive randomized clinical trial in implementation science.

## Appendix

Multimedia Appendix 1 Focus group discussion guide.

Multimedia Appendix 2 Key informant interview guide.

## Abbreviations

ANC: absolute neutrophil count
BDTH: Barau Dikko Teaching Hospital
CBC: complete blood count
CFIR: Consolidated Framework for Implementation Research
FAST: Face drooping, Arm weakness, Speech difficulty, and Time to call for help
Hb: hemoglobin
HU: hydroxyurea
NIH: National Institutes of Health
PedNIHSS: Pediatric National Institutes of Health Stroke Scale
PI: principal investigator
RE-AIM: Reach, Effectiveness, Adoption, Implementation and Maintenance
SCA: sickle cell anemia
SCD: sickle cell disease
SPIN: Stroke Prevention in Nigeria
SPRING trial: Primary Stroke Prevention in Nigeria
TCD: transcranial Doppler
WHO: World Health Organization
YDMH: Yusuf Dantsoho Memorial Hospital
