# Supplementary material for: Primary Prevention of Stroke in Children With Sickle Cell Anemia in Nigeria: Protocol for a Mixed Methods Implementation Study in a Community Hospital
Source: JMIR Res Protoc. 2022 Jun 13;11(6):e37927. doi: 10.2196/37927 (PMC9496111; doi:10.2196/37927)
Supplement: Multimedia Appendix 2 [file resprot_v11i6e37927_app2.docx]

**Multimedia Appendix II.** Key Informant Interview Guide – Hospital Administrators

**Key Informant Biodata:**

**Please kindly provide the following information (circle or tick the most appropriate options).**

1. Age (in years):

2. Gender: □ Male □ Female

3. Marital status: □ Single □ Married □ Widowed □ Divorced □ Separated

4. Tribe: □ Hausa □ Yoruba □ Igbo □ Other (specify)…………..

5. Professional background:

□ Nursing

□ Obstetrician

□ Paediatrician/Neonatologist

□ Medical officer

□ Accountant

□ Administrative staff

□ Other: (please specify):

6. Current position work and location:

7. How many years of experience have you had in this current job?:

8. Can you briefly describe your roles and responsibilities in this position?:

**Thank you for your responses!**

**Facilitator’s welcome, introduction, and instructions to key informant:**

Welcome and thank you for volunteering to take part in this interview. You have been asked to participate as your point of view is important. I realize you are busy, and I appreciate your time.

**Introduction:** This interview is designed to assess your current thoughts and feelings about an intervention that will help in improving the care and management of children with sickle cell disease. This intervention involves the introduction of task shifting in the management of sickle cell disease and stroke in children in your facility. The interview will take no more than forty-five minutes. May I tape the discussion to facilitate its re-collection?

<<*if yes, switch on the recorder*>>

**Anonymity:** Despite being recorded, I would like to assure you that the discussion will be anonymous. The tapes will be kept safely in a locked facility until they are transcribed word for word, then they will be destroyed. The transcribed notes will contain no information that would allow you to be linked to specific statements. You should try to answer and comment as accurately and truthfully as possible. If there are any questions or discussions that you do not wish to answer or participate in, you do not have to do so; however please try to answer and be as frank and involved as possible. You may wish to opt out if you are not interested in participating.

**Ground rules**

- There are no right or wrong answers
- You do not have to speak in any particular order
- When you do have something to say, please do so and stop me to emphasize at any point in time during the interview session.
- Do you have any questions? (*Note them and provide answers as much as possible. Once the respondent agrees, proceed with the interview*)

**Warm up**

First, I’d like you to introduce yourself and what you do. Can you tell me your name?

**Introductory question**

I am just going to give you a couple of minutes to think about your experience of providing care to children and other persons with SCD. Please kindly share your experience with me.

**Guiding questions (based on the CIFR Framework and Survey Objectives)**

1. **Intervention Characteristics**
2. What do you understand about task shifting?
3. How do you feel about task shifting?
4. Do you think task shifting can be done in your institution?
5. In what department or units do you think task-shifting will help?
6. Which cadre of staff would you want to adopt task shifting for?
7. Who do you think should be involved in the decision on task shifting?
8. **Characteristics of the individual** (Knowledge and belief about the intervention)
9. Tell me what you know about sickle cell disease?
10. How do you think SCD affects people? (
    1. *Probe: what problems do you think they have?)*
11. Do you know any children with SCD?
    1. *Probe: describe how he/she looks*
12. Did you know that children with SCD could have stroke?
13. Tell me how you think stroke can affect children and their parents/caregivers?
    1. *Probe*: *intellectually, emotionally, financially*?
14. How do you think you can help children with SCD and their parents?
15. **Outer settings** (Patients’ needs and resources)
16. Tell me the kind of information you think will help you in addressing the needs of patients with SCD and their caregivers in understanding SCD?
17. Tell me in what ways you think this information can best be delivered or disseminated to patients and their caregivers?
18. Tell me more on how the patients can be engaged in understanding SCD and stroke and how it can be prevented.
19. What problems do you foresee will be encountered in the process of engaging patients regarding SCD and stroke?
20. Do you think other health facilities like other PHCs can be involved? Tell me how?
21. **Inner setting** (Implementation climate)
22. Do you think introducing task shifting will help children with SCD in terms of stroke prevention?
23. Tell me how task shifting aligns with your institution’s vision and mission?
24. How will you implement task shifting? How will you form the team? Where will the personnel be selected from? How soon will the team come on board?
25. Tell me how to introduce task shifting?
26. Tell me the resources you will make available for this process?
27. How will the team communicate with the management?
28. Tell me how you will ensure that task shifting is sustained?
    1. *Probe: commitment to project based on role*
29. **Implementation process** (Planning and engaging)
30. How do you intend to start the stroke prevention programme?
31. Tell me the individuals you think are key stakeholders at this time.
    1. Prove: *Are you going to assign? Or allow them to volunteer? What characteristics will you be looking out for?*
32. Tell me how you will ensure staff support the programme.
33. Tell me how you will ensure the programme is continued.
34. How will you get the buy-in of staff within your organisation?
35. Tell me how you will identify champions within the organisation
36. Tell me how you intend to engage them

**Concluding question**

Of all the things we discussed today, what would you say are the most important issues you would like to express about SCD, stroke and task shifting?

**Conclusion**

Thank you for participating. This has been a very useful discussion and we hope you found it interesting. Your opinions will be a very valuable contribution to the study. Please do not hesitate to contact the Principal Investigator or I on any issue related to this subject matter. I would like to remind you that any comments featuring in this report will be anonymous and your privacy will be guarded with utmost confidentiality. Before you leave, please kindly provide me with all information regarding your personal details.

Thank you for your time and attention!
